# Chronic pain in survivors of critical illness: a retrospective analysis of incidence and risk factors

**DOI:** 10.1186/cc12746

**Published:** 2013-05-29

**Authors:** Ceri E Battle, Simon Lovett, Hayley Hutchings

**Affiliations:** 1 School of Medicine, University of Wales Swansea, Swansea, Wales, UK; 2 Physiotherapy Department, Morriston Hospital, Swansea, Wales, UK, This work was completed at the Intensive Care Unit, Morriston Hospital, ABMU Health Board, Swansea, UK

**Keywords:** risk factors, chronic pain, critical illness, retrospective analysis, multivariable analysis

## Abstract

**Introduction:**

Chronic pain has been reported in survivors of critical illness for many years after discharge from hospital. This study investigates the incidence and site of chronic pain in survivors of critical illness between 6 months and 1 year after hospitalization, including ICU admission. A retrospective analysis of the risk factors for chronic pain in this patient group was also completed.

**Methods:**

A questionnaire method was used to investigate the incidence of chronic pain and the specific body parts affected. A retrospective study and multivariable analysis were used to investigate the risk factors for chronic pain in this patient group. All survivors of a general intensive care unit (ICU) in South Wales in a 6-month period were included in this study.

**Results:**

Chronic pain was reported in 44% of all respondents. The shoulder was the most commonly reported joint affected by pain (22%). Risk factors for chronic pain between 6 months and 1 year after ICU discharge were increasing patient age and severe sepsis.

**Conclusions:**

Chronic pain is a problem in survivors of critical illness, especially in the shoulder joint, and further studies are needed investigating therapeutic interventions that address this long-term problem.

## Introduction

Intensive care medicine involves the treatment of critically ill patients with acute physiological derangement and organ failure. Critical illness is now well recognized as being associated with a number of detrimental long-term sequelae that can affect health-related quality of life for up to 5 years after ICU discharge [1]. Limited attention has been placed on the long-term complications of patients discharged from ICU, and mortality or survival rate as an outcome measure has dominated critical care research for decades. More recently, however, patient-centered outcomes are becoming increasingly important [2]. The long-term outcomes now more commonly investigated in critical care research include quality of life, physical fitness, functional capacity, and various psychological factors [3,4].

One aspect of quality of life previously investigated is the experience of bodily pain [4]. Research has highlighted that the majority of patients requiring intensive care will have varying intensities of pain during their stay, but perhaps more important, a recent review reported that this pain commonly persists after discharge [5]. A similar review investigating quality of life after ICU hospitalization reported a higher rate of chronic pain in patients who had been diagnosed with acute respiratory distress syndrome (ARDS) when compared with the matched normal population [6]. Survivors of severe sepsis have also been reported to experience significantly higher levels of pain intensity and pain interference when compared with the normal population [7]. Chronic pain has been reported to be common after surgery, and a number of postoperative risk factors proposed, including psychological vulnerability, anxiety, and depression [8]. A number of longitudinal studies have investigated the length of time patients still experience pain and discomfort after ICU hospitalization, including a recent study that reported that at a mean follow-up of 8 years, pain and discomfort was reported in 57% of patients [9].

The International Association for the Study of Pain (IASP) defines pain as "an unpleasant sensory and emotional experience associated with actual or potential tissue damage" [10]. Pain exceeding the average period of healing of 2 to 3 months and ceasing to serve any apparent protective function is defined as chronic pain [5]. A number of risk factors for chronic pain experience in critical care survivors have been investigated; these include primarily the admission diagnosis, severe sepsis, and ARDS [6,7,11]. The actual body part affected by ongoing pain has not been investigated to date in critical care research.

The first aim of this study, therefore, was to investigate the incidence and site of chronic pain in survivors of critical illness at 6 months after hospitalization, including ICU admission. The second aim of the study was to complete a retrospective analysis of the risk factors for the chronic pain experienced by the survivors of critical illness.

## Materials and methods

### Setting

The study was single centered, based in a large teaching hospital in South Wales (Morriston Hospital). Morriston Hospital admits more than 1,000 patients to the general ICU per year and serves a population of 450,000 people. The general ICU has a broad cross section of specialities, including general medical (35%), surgical (49%), trauma (6%), neurology (4%), and renal (6%). Head-injured patients requiring surgery are transferred to another hospital and were not included in this study. The hospital has a separate burns and cardiothoracic ICU, so these patients were also excluded. No standardized protocol is now used for the assessment and measurement of acute pain on our ICU. Each patient's pain is assessed and managed on an individual basis.

### Sample

All patients who had been admitted between September 2011 and February 2012 were included in this study. These dates were selected so that at least 6 months had passed since the patients had been discharged from ICU. This would ensure that the definition of chronic pain by Kyranou and Puntillo (2012) was accurate and would also provide a sufficient cross section of patients regarding demographics and admission diagnoses [5]. We also wished to include sufficient patients that we could present the unadjusted and adjusted odds ratios and 95% confidence intervals for the risk factors for the development of chronic pain in survivors of critical illness. Peduzzi *et al*. (1995) [12] suggested that the number of patients needed to ensure sufficient power in a retrospective cohort study is equivalent to 10 events per variable (EPV) being investigated [12]. In this study, we set out to investigate 10 variables or risk factors; therefore, a minimum of 100 events (incidence of chronic pain) were required. As a number of studies have suggested that more than 50% of survivors of critical illness have chronic pain, we needed at least 200 patients in total. A 6-month sample provided sufficient patients to achieve this number, allowing for mortality and loss to follow-up.

### Definition of variables

The risk factors under investigation are outlined in Table 2 and were selected *a priori *based on previous research [1,3-9]. This information was obtained from the hospital critical care database "ward-watcher." If the information was missing from the database, the patients' medical notes were retrieved. The Acute Physiology and Chronic Health Evaluation (APACHE II) score is used to predict mortality in ICU patients, and each patient's score was recorded. The diagnosis of severe sepsis was given by using the definition provided by the Surviving Sepsis Campaign (2013) [13]. Patients' primary admission diagnoses were classified as either surgical (including abdominal, maxillofacial, spinal, vascular conditions), general medical (including renal, oncology, respiratory conditions), neurologic (acute and chronic), and trauma. Ventilator days was defined as the number of days the patient required invasive mechanical ventilation. ICU length of stay (ILOS) was defined as the number of whole days the patient was managed on ICU or the high dependency unit (HDU). Hospital length of stay (HLOS) was defined as the total number of days from the day of initial admission until discharge from the hospital.

Any conditions existing before ICU admission that could cause chronic pain were analyzed as a risk factor and labeled together as preexisting conditions. These included chronic pain (reported in any joint), any inflammatory joint condition (osteoarthritis or rheumatoid arthritis), and neurologic conditions (long-term or progressive conditions such as neuralgia, spinal injuries, or multiple sclerosis). Any surgical wounds, injuries/trauma, or ICU interventions that could potentially cause chronic pain (such intercostal chest drains) were also recorded. For the purpose of this study, these were categorized together under the title Wounds.

### Study design

To address both aims of this study, a mixed-methods approach was used: a qualitative design was used in the questionnaire study, and a quantitative design for the retrospective study.

#### Questionnaire study

The first aim was addressed by using a questionnaire and telephone follow-up method. This part of the study was designed by following available guidelines in questionnaire research and the guidelines published in a series of articles in the *British Medical Journal *[14,15]. A short questionnaire was designed for the purpose of investigating incidence of chronic pain, body parts affected and the use of primary healthcare resources to attempt to address the pain. (see Additional file 1) We used the previously validated Brief Pain Inventory method of asking the patient to chart the location of body pain on a body chart [16]. This inventory can be both self-administered and administered over the telephone, as we did in our study. It was also designed for use in patients with chronic diseases and conditions and those with postsurgical pain, a population very similar to our cohort.

As we did not set out to investigate either intensity of pain or the impact of the pain on quality of life, no other validated questionnaire could be identified for use in this study. We were therefore unable to use any other previously validated chronic pain questions. The questions were therefore designed according to guidelines for the assessment of pain in older people from the British Pain Society [17]. These guidelines included using alternative words to describe pain, such as "ache" and also the use of a pain map. Self-report pain questionnaires are reported in these guidelines to have high validity and reliability in older people with no significant cognitive or communication impairment [17].

To assess the clinical sensibility of the questionnaire, it was piloted on a number of ICU survivors attending our follow-up clinic, and, as a result, a number of alterations were made to the questionnaire design. For example, we included a pictorial body chart, which allowed the respondent to highlight the exact body part in which pain was being experienced rather than trying to describe it by using words. Both closed and open-ended questions were used in the questionnaire. The respondents were asked to state whether they were experiencing any ongoing pain since discharge from the ICU (only new pains that they did not have before their ICU admission), and if so, they were requested to describe the body parts affected (either in words or pictorially on the body chart). They were also asked whether they had seen any healthcare professional regarding the described pain.

A preaddressed envelope was included for return of the questionnaire, and all nonresponders were followed up with a telephone call after 2 months, in which the investigator completed the questionnaire according to the patient's responses. The questionnaire responses were entered onto a Microsoft Excel spreadsheet. Any questionnaires with missing demographic data were included in the study, and the remaining responses included in the analysis. If a respondent left blank the question regarding the existence of ongoing pain, this was included in the results as a lack of pain. Response rates were fully recorded, and nonresponder analysis was completed to compare the characteristics of the nonresponders and the responders. Results were presented descriptively by using numbers and percentages. Data analysis was completed by using the Microsoft Excel software. Patients were identified only by their hospital numbers, once completed questionnaires were received.

#### Retrospective study

The hospital database was used to obtain the data required to complete the retrospective study, including all risk factors and outcomes investigated. All data were recorded on a predesigned database. A validation check was completed in which an additional researcher checked the accuracy of data input for 10% of all patients, to reduce information bias. If a patient's notes had missing or incomplete data for the variables under investigation, they were still included in the database. The dataset included demographic variables such as age, gender, and injury mechanism. The independent variables examined were defined *a priori *and consisted of the risk factors for chronic pain highlighted previously in the literature. These included patient age, APACHE II score, primary admission diagnosis, severe sepsis, ventilator days, ILOS, and HLOS. The outcome measure investigated was the incidence of chronic pain.

The issue of missing variables from the hospital database was overcome by retrieving the medical notes for the patient. To ensure confidentiality, patients' names were not recorded during the data-collection period. The dataset was also stored on a hospital-encrypted computer to ensure data security (safe-end protector encryption).

### Ethical approval

A letter explaining the purpose and design of the study was sent to the Chairman of the South West Wales Research Ethics Committee. It was confirmed by the chairman that no ethical approval was required for this study.

### Data analysis

Statistical analyses were performed by using SPSS Version 16 (Chicago) and the R program (version 2.14.1). Patients' demographics were analyzed by using descriptive statistics and presented as numbers and percentages for the categoric variables and means and standard deviations for the continuous variables. For the univariate analysis, Mann-Whitney *U *test was performed for comparisons between the continuous variables (due to lack of normal distribution of these variables) and the Fisher Exact test was performed for each of the independent variables investigated. The same statistical tests were used to complete the nonresponder analysis.

To identify predictors of chronic pain in survivors of critical illness, multivariable analysis was performed. For the categoric variables, the data were coded with a "1" if the variable was present and a "0" if absent. Age, APACHE II score, ventilator days, ILOS, and HLOS were analyzed as continuous variables. For the multivariable analysis, logistic regression with backward stepwise elimination by using the likelihood test statistic was used to assess potential predictors of development of chronic pain in survivors of critical illness. Continuous variables were analyzed as linear terms, as no indication of nonlinearity was found when analyzed by using the multivariable fractional polynomials function in the R program. Adjusted odds ratios and the 95% confidence intervals were calculated for each risk factor. Subgroup analysis was performed by using the same techniques for the most frequently reported body part affected by pain to investigate associated risk factors. Variables were included in the multivariable analysis only if they were statistically significant at *P *< 0.15 in the univariate analysis [18]. Statistical significance for the identification of independent risk factors was set at *P *< 0.05.

## Results

**Figure 1:**
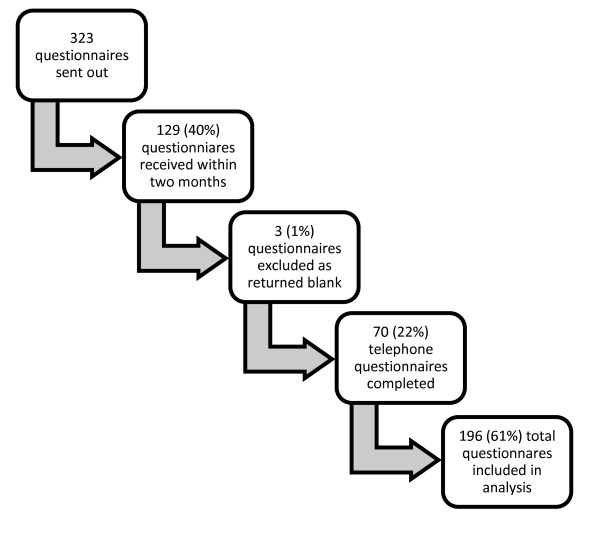
**Diagram illustrating response rate**.

Figure 1 outlines the response rate in the questionnaire study. In total, 404 patients were identified as having an ICU admission in the 6-month period investigated. As 81 patients had died, 323 patients were included in the study. Nonresponder analysis revealed no statistical differences in any of the demographic variables under investigation. Table 1 outlines the characteristics of the nonresponders compared with the responders. Only three questionnaires were excluded, as they were returned with no responses.

**Table 1:** Nonresponder analysis: comparison of demographics and risk factors between responders and nonresponders

Variable	Responders *n *= 196	Nonresponders *n *= 208	*P *value
Male	106	115	0.84

Female	90	93	0.84

Sepsis	43	49	0.72

Medical	75	75	0.68

Surgical	101	109	0.92

Neurology	7	9	0.80

Trauma	13	15	0.85

Preexisting conditions	20	31	0.18

Wounds	119	136	0.35

Mean age	61	63	0.76

Mean APACHE II	15	16	0.42

Mean number of ventilator days	2.1	3.4	0.72

Mean number of ILOS	6.2	7.9	0.86

Mean number of HLOS	17.8	20.6	0.96

HLOS, total hospital length of stay; ILOS, ICU length of stay.

**Table 2:** Results obtained from the questionnaire responses: incidence of chronic pain, body parts affected, and healthcare input

Response	*N *= 196	%
Total number of patients reporting no pain	110	56

Total number of patients reporting pain	86	44
Shoulder	44	22
Lower limb	17	9
Lumbar spine	17	9
Cervical spine	12	6
Upper limb	12	6
Abdomen	8	4
Pelvis	6	3
Thorax	2	1
Nonspecific (all-over ache)	4	2

Total number of patients who have received healthcare input specifically for pain	62	32

Table 2 outlines the responses obtained from the questionnaire study. In total, 86 patients (44%) reported still experiencing pain at least 6 months after ICU discharge, with 62 patients (32%) reporting the use of a healthcare professional to attempt to address the described pain. The most frequently reported body part affected was the shoulder (22%).

The results of the retrospective study are outlined in Table 3. The number and percentages or means and standard deviations are presented for each variable investigated. Table 2 also illustrates the statistically significant risk factors (*P *< 0.05) for the incidence of chronic pain at 6 months to 1 year after ICU discharge by using univariate analysis. Unadjusted odds ratios and 95% confidence intervals are included for the categoric variables. Significant risk factors

**Table 3:** Comparison of the risk factors for the two groups: results of the univariate analysis

	Pain group	*n *= 86	No-pain group	*n *= 110		
**Categoric variables**	***n***	**%**	***n***	**%**	***P *value **	**Unadjusted OR (95% CI)**

Male	49	57	57	52	0.56	1.2 (0.7-2.2)
Female	37	43	53	48	0.56	1.2 (0.7-2.2)
Sepsis	31	36	13	12	0.0001*^a^*	4.2 (2.0-8.7)
Medical	37	43	38	35	0.23	1.4 (0.8-2.6)
Surgical	38	44	63	57	0.08	0.6 (0.3-1.0)
Neurology	3	3	4	4	1.00	0.9 (0.2-4.4)
Trauma	8	9	5	5	0.24	2.2 (0.7-6.8)
Preexisting conditions	8	9	12	11	0.81	0.8 (0.3-2.2)
Wounds	45	52	74	67	0.04*^a^*	0.5 (0.3-1.0)

**Continuous variables**	**Mean**	**SD**	**Mean**	**SD**	***P *value**	

Mean age	65	16.8	61	18.8	0.07	
Mean APACHE II score	16	6.5	15	5.4	0.43	
Mean number ventilator days	5	9.4	2	4.2	0.36	
Mean ILOS	10	11.7	6	6.2	0.01*^a^*	
Mean HLOS	24	21.7	18	13.2	0.07	

CI, confidence interval; HLOS, hospital length of stay; ILOS, ICU length of stay;

OR, odds ratio. ^a^Significant at *P *< 0.05.

Table 4 highlights the results of the multivariable analysis. All risk factors with a *P *< 0.15 on univariate analysis were included in the multivariable analysis. These variables were sepsis, age, wound, ICU and hospital length of stay, and a primary admission diagnosis of surgery. Significant risk factors for chronic pain at 6 months to 1 year after ICU discharge include patient age and presence of severe sepsis during ICU admission. Adjusted odds ratios and the 95% confidence intervals are presented.

**Table 4:** Risk factors for the incidence of chronic pain after ICU discharge: results of the multivariable analysis

Risk factor	*P *value	Adjusted OR (95% CI)
Age	0.025*^a ^*	1.0 (1.00-1.04)

Sepsis	0.001*^a ^*	4.3 (2.04-9.23)

Wound	0.075	0.6 (0.31-1.06)

ILOS	0.347	1.0 (0.98-1.06)

HLOS	0.707	1.0 (0.98-1.03)

Surgical	0.447	0.7 (0.36-1.58)

CI, confidence interval; HLOS, hospital length of stay; ILOS, ICU length of stay;

OR, odds ratio. ^a^Significant at *P *< 0.05.

Table 5 highlights the results of the subgroup analysis, investigating the risk factors for shoulder pain at 6 months to 1 year after ICU discharge. Shoulder pain has been selected for further analysis as a result of its high incidence. Adjusted odds ratios and the 95% confidence intervals are presented. The significant risk factors for shoulder pain were severe sepsis and hospital length of stay.

**Table 5:** Risk factors for the incidence of shoulder pain after ICU discharge: results of the multivariable analysis

Risk factor	*P *value	Adjusted OR (95% CI)
HLOS	0.026*^a^*	1.0 (1.00-1.04)

Sepsis	0.001*^a^*	4.1 (1.90-8.83)

ILOS	0.670	1.0 (0.96-1.07)

Age	0.643	1.0 (0.98-1.03)

Surgical	0.708	1.2 (0.48-2.98)

CI, confidence interval; HLOS, hospital length of stay; ILOS, ICU length of stay;

OR, odds ratio. ^a^Significant at *P *< 0.05.

## Discussion

Chronic pain is increasingly recognized as a problem in survivors of critical illness. Despite an increased awareness of its contribution to reduced quality of life, pain remains a significant problem for survivors of critical illness [5]. Conflicting evidence exists regarding the incidence of chronic pain in this patient group. The results of this study, however, highlight that nearly half of all respondents still experience pain at least 6 months after ICU discharge. This concurs with a previous study that reported that 56% of patients still reported pain at 2 years after ICU discharge [3]. In a Dutch study by Hofhuis *et al.*, 2008 [19], health- related quality of life was reported to be significantly lower in critically ill patients at pre-ICU admission when compared with the healthy Dutch population. They also reported that health-related quality of life remained significantly lower than that of the healthy Dutch population at 6 months after ICU discharge, except in the bodily pain score, which does not concur with the results of this study [19].

This study reported that more than 20% of patients were experiencing shoulder pain at least 6 months after ICU discharge. This result is higher than that of an age-matched general population of 15% in a study in the Netherlands in 2011 and 11.7% in a British study in 2002 [20,21]. In a survey of chronic pain in Europe and Israel, chronic shoulder pain was reported in 9% of the population [22]. In a recent small study completed in England in 2012, 80% of ICU survivors were reported to have shoulder dysfunction over a period of 1 year after ICU discharge [23]. Limited previous research exists investigating specific body parts affected by pain in survivors of critical illness. A possible contributory factor for the incidence of shoulder pain may include the patient's reluctance or inability to move the shoulder girdle because of the position of the central line, dialysis lines, and ventilator tubing. Another possible cause of shoulder pain is the lack of muscle tone during critical illness. The shoulder joint is well recognized as an unstable joint when lacking muscle tone [24], and this may lead to chronic pain in survivors of critical illness. One reason for this is that the shoulder is potentially put under undue strain during frequently performed nursing procedures, such as rolling, at a time when it is at its most vulnerable. An increased awareness of the incidence of shoulder pain in patients discharged from ICU should encourage healthcare professionals responsible for caring for these patients to take appropriate measures to handle the shoulder joint appropriately at all times.

Nearly one third of all patients reported using healthcare resources in an attempt to address their chronic pain, which has potential cost implications for provision of ongoing care for these patients. In the survey by Breivik *et al*. (2006) [22], 60% of patients with chronic pain had visited their GP about their pain two to nine times in the last 6 months [22]. In a study by Der Schaaf *et al*. (2009) [11], it was reported that at 3 months after hospital discharge, 45% of all patients were following an interdisciplinary rehabilitation program to address their ongoing functional needs [11]. Recent emphasis has been placed on the need for follow-up of this patient group and the provision of rehabilitation programs (NICE 2009), and our study's reports supports these recommendations [25].

No significant differences were found between the patients with chronic pain and those without, in terms of gender, primary admission criteria, or APACHE II scores. Dowdy *et al*. (2005) [4] similarly concluded in their systematic review that gender and admission diagnosis were not predictors of quality of life. Dowdy *et al*. (2005) [4] did report, however, that a trauma diagnosis was a predictor of pain; however, because of the small number of trauma patients in this study, cross-comparison is not possible.

The significant risk factors for chronic pain on the univariate analysis were severe sepsis and ICU length of stay. Primary admission diagnosis of surgery, wound, ICU and hospital length of stay were dropped as significant risk factors in the multivariable analysis, and this may reflect the influence of confounding in an observational study of this design. In a study by Timmers *et al*. (2011) [9], ICU length of stay and mechanical ventilation days were also not reported to be significant risk factors for chronic pain after discharge from ICU, which concurs with the results of the multivariable analysis in this study.

On multivariate analysis, one of the significant risk factors for chronic pain was severe sepsis. These results concur with a German study by Zimmer *et al*. (2006) [7], in which patients who had survived severe sepsis were also reported to have significantly higher levels of pain, although the time since discharge is unclear. One potential explanation for this reported chronic pain was suggested by Zimmer *et al*. (2006) [7], who described the proinflammatory cytokine response that has been demonstrated to increase pain intensity. Another possible explanation is that the patient with severe sepsis often requires high levels of inotropic support, thus contraindicating early mobilization and rehabilitation. Further prospective studies are required to investigate possible mechanisms for the influence of severe sepsis on chronic pain.

The patient's age was another significant risk factor for chronic pain in patients discharged from ICU. This may be due to the normal physiologic changes associated with increased age, such as reduced muscle mass, reduced levels of blood and tissue metabolites, and a poor nutritional status. A number of studies have reported that age influences the patient's functional recovery, and these are summarized in the review by Dowdy *et al*. (2005) [4]. In contrast to the results of this study, however, Dowdy *et al*. (2005) reported that age did not influence pain experience at 6 months after ICU discharge [4].

Younger age has been reported as a risk factor in investigations of chronic pain after surgery; however, these results remain inconsistent [26]. A number of mechanisms for this have been proposed and relate primarily to reduction in peripheral nociceptive function with increased age [26]. In contrast to the postsurgery studies, increased age may be a risk factor for chronic pain in post-ICU patients because of a number of other potential mechanisms. These include the normal physiologic ageing processes affecting the musculoskeletal system, an increased number of comorbidities, and higher rates of polypharmacy evident in the elderly population. In this study, presence of a surgical wound was not a risk factor for chronic pain on multivariable analysis.

This study highlighted the incidence of chronic pain and its potential risk factors at 6 months to 1 year after ICU discharge. One interesting finding of this study was that the existence of preexisting chronic pain conditions before ICU admission was not a risk factor for chronic pain after discharge from the ICU. An increased awareness of the incidence of chronic pain should ultimately result in an attempt to preempt it, through the aggressive use of therapeutic interventions for pain. Early mobilisation and rehabilitation during the ICU stay is increasingly considered one of the most effective strategies for reducing pain and functional disability after discharge, but further research is still needed [27]. Simply having an understanding of the potential causes of ongoing shoulder pain and how to minimize the risk of its occurrence, such as appropriate handling of the joint, could improve long-term outcomes in this patient group.

The use of ICU follow-up clinics was recommended by the National Institute of Clinical Excellence (NICE) in 2009, and these clinics provide the opportunity to address ongoing pain and dysfunction [25]. Further prospective studies are needed investigating long-term outcomes in survivors of critical illness and possible therapeutic interventions to reduce chronic pain.

The present study has a number of limitations. As a result of the study design and the inherent nature of patients, a number of the risk factors investigated were potentially interdependent, so an increase in one variable inadvertently results in an increase in another. Multivariable logistic regression with backward elimination techniques was used to address this issue of collinearity. In clinical research, however, this is difficult to overcome because of the nature of the study population, and therefore, the results of this study should be interpreted with this in mind.

A 6- month cohort was used to include a sufficient number of patients to provide valuable results. It could be suggested that a patient's pain symptoms may potentially change over the 6- month time period used in this study; however, in a study of this nature, this is unavoidable. It is also questionable as to how significantly a patient's pain symptoms would change over a 6-month period, because of the nature of chronic pain. One method of overcoming this would be to complete a multicentered study over a shorter time period.

A sample size of 100 patients with chronic pain was needed for this study according to Peduzzi *et al*. (1995) [12], but as only six variables were entered into the final multivariable analysis, the sample size was considered sufficient for the final analysis. The low response rate (61%) may have introduced nonresponse bias; however, nonresponder analysis was used in an attempt to control for this bias. The use of interviews to complete the questionnaires and the methods used to handle missing data in this study may also have inadvertently led to a degree of interviewer or interpretation bias. As a result of these different types of potential bias, the results of this study should be interpreted with caution.

No suitable previously validated questionnaire was available for use in this study. Therefore, a further limitation of this study was the use of a newly designed questionnaire. The study results may lack validity and generalizability as a result. The questionnaire was piloted on a number of survivors of critical illness to help to overcome this limitation. Another limitation of investigating outcomes in survivors of critical illness is the high mortality rate and loss to follow-up. This may introduce a degree of reporting bias, but this is unavoidable in a study of this type.

In an observational study using a retrospective database analysis, the potential exists for information bias. Data may have been incorrectly entered into the database at the time of the patient's admission, or the data may be incorrectly copied for use in the study. A validation check was used in an attempt to overcome this error of data extraction and data input and thus reduce information bias. A further limitation is the potential underreporting of severe sepsis by doctors in ICU. The aim initially was also to include ARDS in our study, but because of the obvious underreporting of ARDS data, the decision was made to exclude ARDS as a risk factor from our study. Availability of further information regarding other factors important to pain experience (for example, medications used during the ICU stay) would have improved the reliability and validity of this study. A further prospective study would be needed to overcome these limitations, and this should be considered when interpreting the study results.

## Conclusions

In summary, two main conclusions can be drawn from this study. A high incidence of chronic pain is found in survivors of critical illness at 6 months to 1 year after ICU discharge. The risk factors for the pain experienced are increasing age and severe sepsis. These results concur with the findings of a number of previous studies but also highlight areas for further research. Potentially beneficial research would include studies that investigate various therapeutic interventions to prevent this chronic pain experienced by patients discharged from ICU. An awareness of the risk factors for the onset of chronic pain allows the healthcare professionals caring for the patient potentially to address contributing factors, such as careful handling of the shoulder in paralyzed or elderly sepsis patients.

## Key messages

• Chronic pain was reported by 44% of patients 6 months to 1 year after ICU discharge

• Shoulder pain was reported by 22% of patients 6 months to 1 year after ICU discharge

• Of patients discharged from an ICU, 32% seek further input from healthcare professionals for chronic pain.

• Severe sepsis and increasing patient age were the significant risk factors for chronic pain in this patient group.

• Further prospective studies are needed to investigate therapeutic interventions to reduce the incidence of chronic pain in survivors of critical illness.

## Abbreviations

APACHE: Acute Physiology and Chronic Health Evaluation; ARDS: acute respiratory distress syndrome; CI: confidence interval; HLOS: hospital length of stay; ICU: intensive care unit; ILOS: intensive care unit length of stay; NICE: National Institute of Clinical Excellence; OR: odds ratio.

## Competing interests

The authors declare that they have no competing interests (see Additional file 2).

## Authors' contributions

The study was conceived and designed by CB and HH. CB supervised the conduct of the trial. HH and SL provided the statistical advice, and CB and SL analyzed the data. CB drafted the manuscript, and HH and SL contributed substantially to its revision. CB takes overall responsibility for the article. All authors read and approved the final version of the manuscript.

## References

[B1] Cuthbertson BH Roughton S Jenkinson D MacLennan G Vale L Quality of life in the five years after intensive care: a cohort study Crit Care 2010 17 R6 10.1186/cc8848 20089197 PMC2875518

[B2] Modrykamien AM The ICU follow up clinic: a new paradigm for intensivists Respir Care 2012 17 764 772 22152275 10.4187/respcare.01461

[B3] Jacodic HK Jacodic K Podbregar M Long term outcome and quality of life of patients treated in a surgical intensive care: a comparison between sepsis and trauma Crit Care 2006 17 R134 10.1186/cc5047 16978417 PMC1751058

[B4] Dowdy DW Eid MP Sedrakyan A Mendez-Tellez PA Pronovost PJ Herridge MS Needham DM Quality of life in adult survivors of critical illness: a systematic review Intensive Care Med 2005 17 611 620 10.1007/s00134-005-2592-6 15803303

[B5] Kyranou M Puntillo K The transition from acute to chronic pain: might intensive care unit patients be at risk? Ann Intensive Care 2012 17 36 http://annalsofintensivecare.com/content/2/1/36 10.1186/2110-5820-2-36 22898192 PMC3488025

[B6] Dowdy DW Eid MP Dennison CR Mendez-Tellez PA Herridge MS Guallar E Pronovost PJ Needham DM Quality of life after acute respiratory distress syndrome: a meta-analysis Intensive Care Med 2006 17 1115 1124 10.1007/s00134-006-0217-3 16783553

[B7] Zimmer A Rothaug J Mescha S Reinhart K Meissner W Marx G Chronic pain after surviving sepsis Crit Care 2006 17 suppl 1 421 10.1186/cc476816959049

[B8] Perkins FM Kehlet H Chronic pain as an outcome of surgery Anaesthesiology 2000 17 1123 1133 10.1097/00000542-200010000-00038 11020770

[B9] Timmers TK Verhofstad MH Moons KH van Beeck EF Leenen LP Long-term quality of life after surgical intensive care admission Arch Surg 2011 17 412 418 10.1001/archsurg.2010.279 21173281

[B10] International Association for the study of Pain Taxonomy http://www.iasp-pain.org/AM/Template.cfm?Section=Pain_Definitions

[B11] Der Schaaf M Beelan A Dongelmans D Vroom M Nollet F Poor functional recovery after critical illness: a longitudinal study J Rehabil Med 2009 17 1041 1048 10.2340/16501977-0443 19893999

[B12] Peduzzi P Concato J Feinstein AR Holford TR Importance of events per independent variable in proportional hazards regression analysis J Clin Epidemiol 1995 17 1503 1510 10.1016/0895-4356(95)00048-8 8543964

[B13] Dellinger RP Levy MM Rhodes A Annane D Gerlach H Opal SM International Guidelines for Management of Severe Sepsis and Septic Shock: 2012 Crit Care Med 2013 17 580 637 http://www.sccm.org/Documents/SSC-Guidelines.pdf 10.1097/CCM.0b013e31827e83af 23353941

[B14] Boyton PM Administering, analysing and reporting your questionnaire BMJ 2004 17 1371 1375 10.1136/bmj.328.7452.1371 PMC420299 15178620

[B15] Boyton PM Greenhalgh T Selecting, designing and developing your questionnaire BMJ 2004 17 1312 1315 10.1136/bmj.328.7451.1312 15166072 PMC420179

[B16] Cleeland CS Chapman CR, Loeser JD Measurement of pain by subjective report Advances in Pain Research and Therapy: Issues in Pain Measurement 1989 17 New York: Raven Press 391 403

[B17] Royal College of Physicians, British Geriatrics Society and British Pain Society The assessment of pain in older people: national guidelines: concise guidance to good practice series 2007 No 8. London: RCP

[B18] Sauerbrei W Meier-Hirmer C Benner A Royston P Multivariable regression model building by using fractional polynomials: description of SAS, STATA and R programs Comput Stat Data Analysis 2006 17 3464 3485 10.1016/j.csda.2005.07.015

[B19] Hofhuis JG Spronk PE van Stel HF Schrijvers GJ Rommes JH Bakker J The impact of critical illness on perceived health-related quality of life during ICU treatment, hospital stay, and after hospital discharge: a long-term follow up study Chest 2008 17 377 385 10.1378/chest.07-1217 17925419

[B20] Bekkering GE Bala MM Reid K Kellen E Harker J Riemsma R Huygen FJ Kleijnen J Epidemiology of chronic pain and its treatment in the Netherlands Neth J Med 2011 17 141 153 21444943

[B21] Badcock LJ Lewis M Hay EM McCarney R Croft PR Chronic shoulder pain in the community: a syndrome of disability or distress? Ann Rheum Dis 2002 17 128 131 10.1136/ard.61.2.128 11796398 PMC1754001

[B22] Breivik H Collett B Ventafridda V Cohen R Gallacher D Survey of chronic pain in Europe: prevalence, impact on daily life and treatment Eur J Pain 2006 17 287 333 10.1016/j.ejpain.2005.06.009 16095934

[B23] Gustafson O The incidence of shoulder dysfunction in ICU survivors [Abstract]

[B24] Labriola JE Lee TQ Debski RE McMahon PJ Stability and instability of the glenohumeral joint: the role of shoulder muscles J Shoulder Elbow Surg 2005 17 32 38 10.1016/j.jse.2004.09.014 15726085

[B25] National Institute for Clinical Excellence CG83: Critical Illness Rehabilitation: Guideline 2009 http://www.nice.org.uk/nicemedia/live/12137/58250/58250.pdf

[B26] Bruce J Quinlan J Chronic post-surgical pain Br J Pain 2011 17 23 29 10.1177/204946371100500306 PMC4590073 26526062

[B27] Adler J Malone D Early mobilisation in the intensive care unit: a systematic review Cardiopulm Phys Ther J 2012 17 5 13 22807649 PMC3286494

